# Type III secretion system 1 genes in *Vibrio parahaemolyticus* are positively regulated by ExsA and negatively regulated by ExsD

**DOI:** 10.1111/j.1365-2958.2008.06326.x

**Published:** 2008-06-16

**Authors:** Xiaohui Zhou, Devendra H Shah, Michael E Konkel, Douglas R Call

**Affiliations:** 1 Department of Veterinary Microbiology and Pathology, Washington State UniversityPullman, WA, USA; 2 School of Molecular Biosciences, Washington State UniversityPullman, WA, USA

## Abstract

*Vibrio parahaemolyticus* harbours two distinct type III secretion systems (T3SS1 and T3SS2). A subset of 10 T3SS1 genes are transcribed when *V. parahaemolyticus* is grown in tissue culture medium [Dulbecco's modified Eagle's medium (DMEM)], while transcription of these genes (except *exsD*) is minimal upon growth in Luria–Bertani-Salt (LB-S). Transcription of T3SS1 genes and cytotoxicity towards HeLa cells was prevented by deletion of *exsA* while complementation with *exsA* restored these traits. Overexpression of ExsA in the wild-type strain, NY-4, activated the transcription of T3SS1 genes when bacteria were grown in LB-S. Thus, ExsA is necessary and sufficient to induce the transcription of T3SS1 genes. Deletion of the *exsD* permitted the transcription of T3SS1 genes when bacteria were grown in the LB-S medium and complementation with the wild-type *exsD* gene-blocked transcription of T3SS1 genes. Overexpression of ExsD in NY-4 prevented the transcription of T3SS1 gene when bacteria were grown in DMEM. A gel mobility shift assay demonstrated that purified ExsA protein binds a novel motif in the upstream region of *vp1668* and *vp1687,* indicating that ExsA interacts directly with the promoter sequences of T3SS1 genes. ExsA positively regulates the expression and secretion of Vp1656 while ExsD negatively regulates the expression and secretion of Vp1656.

## Introduction

*Vibrio parahaemolyticus* is a Gram-negative, halophilic pathogen that is commonly associated with consumption of raw or undercooked seafood (Joseph *et al*., 1982). Infection with *V. parahaemolyticus* results in diarrhoea, nausea, vomiting, headache, fever and chills. In addition to typical gastrointestinal infections, *c.* 5% of *V. parahaemolyticus* infections advance to septicemia (Hlady and Klontz, 1996) and these infections may be fatal, especially in immunocompromised patients or those with pre-existing medical conditions, such as liver disease or diabetes (Yeung and Boor, 2004).

Thermostable direct haemolysin (TDH) is a major virulence factor of *V. parahaemolyticus*. TDH forms pores in red blood cells, causes increased short circuit current, increases Cl^-^ secretion in human colonic epithelia cells and enhances Ca^2+^ entry from the extracellular medium (Takahashi *et al*., 2001). TDH-related haemolysin is another toxin (Sochard and Colwell, 1977) that is heat-labile and also induces Ca^2+^-activated Cl^-^ channel leading to altered ion flux and secretory diarrhoea (Takahashi *et al*., 2001). A *tdh* deletion mutant has reduced the ability to cause fluid accumulation in ileal loops of a rabbit model (Lin *et al*., 1993). In contrast, Park *et al*. (2004) recently demonstrated that a *tdh* deletion mutant retains the ability to cause fluid accumulation. Furthermore, both *V. parahaemolyticus* TDH-positive and -negative strains disrupt epithelial tight junctions, possibly resulting in the dissemination of bacteria into the host circulation system (Lynch *et al*., 2005). These studies indicate that there are factors, in addition to TDH, that contribute to the pathogenesis of *V. parahaemolyticus*. Some of these include cell-associated haemagglutinin (Nagayama *et al*., 1994), pili (Nakasone and Iwanaga, 1990, 1992), vibrioferrin (Dai *et al*., 1992) and ToxR (Lin *et al*., 1993).

Type III secretion systems (T3SSs) enable bacteria to translocate proteins directly into host cells where they interfere with normal cellular functions (Cornelis and Van Gijsegem, 2000; Cornelis, 2006). T3SSs are divided into three parts: the secretion apparatus, translocators and effectors (Hueck, 1998). The secretion apparatus forms an injector complex in the surface of Gram-negative bacteria. The translocator is a needle structure, which when inserted into the membrane of host cells, allows the effectors to be transported through the cell membrane into the cytosol or other compartments of host cells (Hueck, 1998). While genes encoding the secretion apparatus are conserved among different bacteria, genes encoding effectors can be unique to different organisms (Troisfontaines and Cornelis, 2005).

The genome sequence of *V. parahaemolyticus* shows that this organism harbours two distinct T3SSs encoded in chromosomes 1 (T3SS1) and 2 (T3SS2) (Makino *et al*., 2003). T3SS1 is similar to the Ysc secretion system in *Yersinia* and the T3SS2 is similar to the Inv-Mxi-Spa secretion system in *Salmonella* and *Shigella* (Troisfontaines and Cornelis, 2005). The Ysc secretion system is typically associated with cytotoxicity while the Inv-Mxi-Spa secretion system usually contributes to host cell invasion (Troisfontaines and Cornelis, 2005). T3SS1 of *V. parahaemolyticus* induces host cell death characterized by cell swelling, vacuole formation in the cytosol and pore formation in the membrane of host cells that is caspase-independent (X. Zhou, M.E. Konkel, and D.R. Call, submitted). T3SS2 appears to be involved in the intestinal fluid accumulation (Park *et al*., 2004).

Secretion of effector proteins is stimulated by contact with host cells, but secretion can also be induced by growing bacteria under suitable conditions (Hueck, 1998). For example, the T3SSs of *Pseudomonas* and *Yersinia* can be activated by growing in media containing a chelator, such as nitriloacetate or ethylene glycol tetraacetic acid, which is conventionally referred to as low-calcium media (Straley *et al*., 1993; Vallis *et al*., 1999). Activation of the T3SS of *Shigella* is induced by culturing bacteria in media containing Congo red (Bahrani *et al*., 1997). The mechanism of transcriptional control of T3SS genes in some bacteria has been described. For example, expression of *Pseudomonas* T3SS genes in low-calcium media is controlled at the transcriotional level by an AraC-like transcriptional activator, ExsA (Yahr *et al*., 1995). ExsA binds to a consensus sequence (TNAAANA) approximately 50 bp pairs upstream of the transcriptional start site of the T3SS genes, leading to the transcription of T3SS genes (Hovey and Frank, 1995). ExsA also binds a negative regulator, ExsD, whose overexpression inhibits T3SS gene transcription (McCaw *et al*., 2002).

The T3SS1 of *V. parahaemolyticus* is composed of *c.* 42 genes (*vp1656–vp1697*) of which 30 genes have sequence similarity with genes encoding the apparatus in *Yersinia* while the remaining 12 open reading frames are hypothetical genes and may encode effectors proteins. It is unclear how the transcriptional activation of these 42 genes is controlled. At the terminus of the T3SS1 gene cluster, there are two genes, *vp1699* and *vp1698*, that share sequence similarity with the *Pseudomonas* genes *exsA (*40%) and *exsD* (30%) respectively. We hypothesize that the *V. parahaemolyticus vp1699* and *vp1698*, designated from this point forward as *exsA* and *exsD*, serve as transcriptional regulators in *V. parahaemolyticus.* In this study, we examined the conditions that induce the transcription of T3SS genes and demonstrate how ExsA and ExsD regulate transcription of T3SS1 genes.

## Results

### T3SS1 genes of *V. parahaemolyticus* are transcribed in Dulbecco's modified Eagle's medium, but not transcribed in Luria–Bertani-salt media

**Fig. 1:**
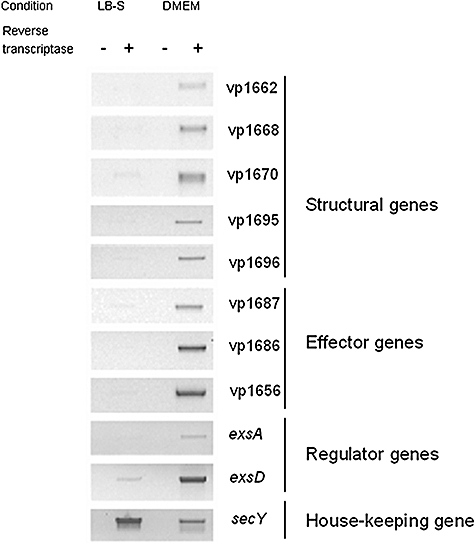
RT-PCR showing expression of several apparatus, effector and hypothetical regulatory genes from T3SS1. cDNA (+) made from total RNA isolated from *V. parahaemolyticus* strain NY-4 under different conditions (LB-S, DMEM) was used as template for PCR. Mock reactions (−), which did not contain reverse transcriptase in the RT reaction, were used as controls against genomic contamination of the RNA preparations. *SecY* was a house-keeping gene used as an internal control to ensure that RNA was present in all samples.

*Vibrio parahaemolyticus* was grown in Luria–Bertani (LB) medium supplemented with 2.5% sodium chloride [LB-salt (LB-S)] and Dulbecco's modified Eagle's medium (DMEM) supplemented with 1% FBS. We selected 10 T3SS1 genes from the T3SS1 gene cluster to monitor gene expression by RT-PCR. All 10 genes were transcribed when bacterial were grown in DMEM (Fig. 1). Only *exsD* was clearly transcribed when bacterial were grown in LB-S (Fig. 1). *Vp1656*, *vp1670*, *vp1687* and *vp1696* were very weakly transcribed (faint bands) when bacteria were grown in LB-S (Fig. 1). Transcription of the house-keeping gene, *secY*, was similar for all culture conditions. In the absence of reverse transcriptase, PCR results were negative, indicating no DNA contamination for these samples. These results demonstrated that T3SS1 genes of *V. parahaemolyticus* are transcribed when cultured in DMEM. Hereafter we refer to DMEM and LB-S as the inducing condition and non-inducing condition, respectively, for the transcription of T3SS1 genes.

### ExsA is required for cytotoxicity induced by wild-type *V. parahaemolyticus*

**Fig. 2:**
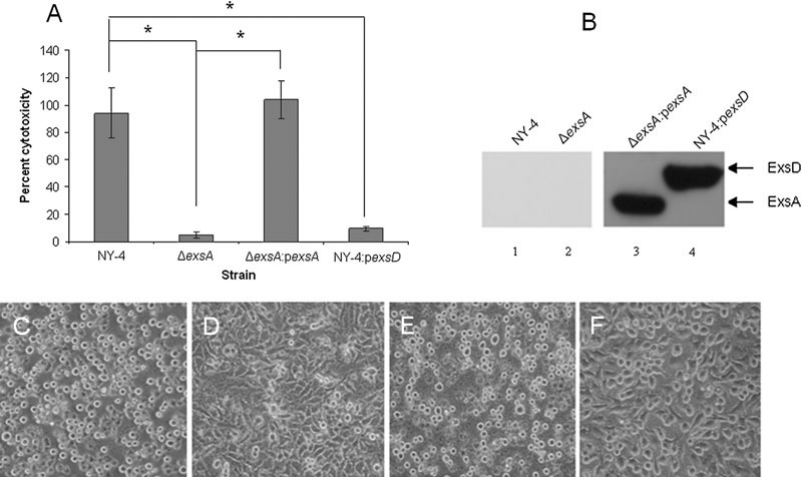
Mutation of *exsA* gene and overexpression of *exsD* gene in wild-type strains attenuated the ability of *V. parahaemolyticus* to induce host cell death. A. HeLa cells were lysed by wild-type (NY-4) and *exsA* complement (Δ*exsA* : p*exsA*) strains of *V. parahaemolyticus* while strain Δ*exsA* and wild-type strain transformed with *exsD* plasmid (NY-4 : p*exsD*) had significantly lower levels of cytotoxicity against HeLa cells (bars = standard deviation for three replicates; star represents statistical difference *P* < 0.05). B. Expression of *exsA* and *exsD* in Δ*exsA* and wild-type strains respectively. Anti-His antibody detected the ExsA and ExsD in the strains of Δ*exsA* : p*exsA* and NY-4 : p*exsD* respectively (Lanes 3 and 4), but not in the wild-type or Δ*exsA* controls. Morphological observations under light microscope for HeLa cells infected with (C) NY-4 strain, (D) Δ*exsA* strain, (E) Δ*exsA* : p*exsA* strain and (F) NY-4 : p*exsD* strain.

Because ExsA is a positive regulator of T3SS genes in *Pseudomonas*, we determined if an *exsA* knockout is attenuated for cytotoxicity. Cytotoxicity was determined by measuring the lactate dehydrogenase (LDH) release by HeLa cells after infection with *V. parahaemolyticus* strains. Four hours after infection with the wild-type NY-4 strain, *c.* 95% of the HeLa cells were lysed, which is consistent with a previous report (X. Zhou, M.E. Konkel, and D.R. Call, submitted), while the Δ*exsA* strain induced less than 5% of HeLa cell death (Fig. 2A). HeLa cells infected with wild-type strain appeared rounded (Fig. 2C) while HeLa cell infected with Δ*exsA* strain were similar to uninfected cells (Fig. D and data not shown). To avoid the possibility that deletion of *exsA* had polar effects, we ectopically expressed ExsA-6xHis in a plasmid and measured if cytotoxicity was restored. Western blot showed that the predicted protein was expressed in Δ*exsA* complemented with an *exsA* plasmid (Fig. 2B, lane 3) and the complemented strain recovered the full phenotype with *c.*100% of the HeLa cells lysed after infection (Fig. 2A). Cell rounding was also restored when HeLa cells were infected with complemented strain (Fig. 2E). These results demonstrated that ExsA is required for *V. parahaemolyticus* to induce host cell death.

### ExsA is required for the transcription of T3SS1 genes in DMEM and is sufficient to activate the transcription of T3SS1 genes in LB-S medium

**Fig. 3:**
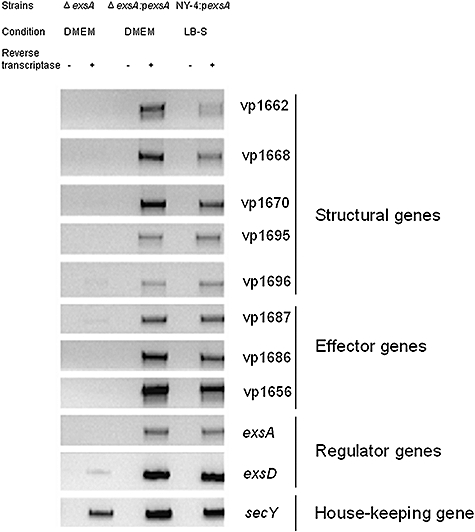
RT-PCR showing expression of T3SS1 genes and *secY*. cDNA (+) made from total RNA isolated from Δ*exsA*, Δ*exsA* : p*exsA* strains under the inducing (DMEM) growth condition, and NY-4 : p*exsA* strains under non-inducing (LB-S) growth condition was used as template for the RT reactions. Mock reactions (−), which did not contain reverse transcriptase, were used as controls against genomic contamination of the RNA preparations.

Deletion of the *exsA* eliminated transcription of T3SS1 genes in the DMEM while expression of ExsA ectopically from a plasmid restored the transcription of T3SS1 genes (Fig. 3), indicating that ExsA is necessary for the transcription of T3SS1 genes in DMEM. Ectopic expression of ExsA in the wild-type strain permitted the transcription of T3SS1 genes even when bacteria were grown in non-inducing medium (i.e. LB-S) (Fig. 3), indicating that ExsA is sufficient to activate the transcription of T3SS1 genes even when bacterial were grown in non-inducing conditions. These results also indicate that T3SS1 transcriptional activation by environmental signals requires transcription of *exsA*.

### ExsD is a negative regulator of T3SS1 genes of *V. parahaemolyticus*

**Fig. 4:**
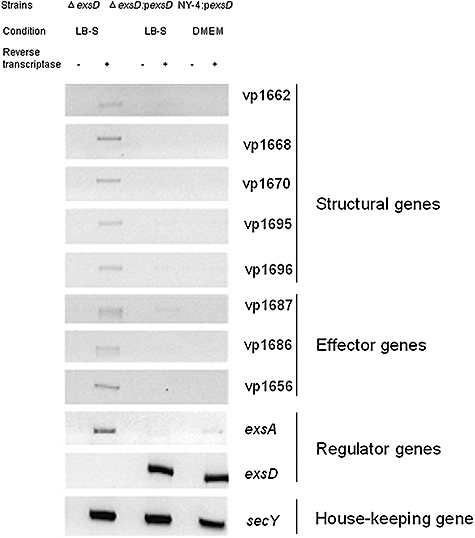
RT-PCR showing expression of T3SS1 genes and *secY.* cDNA (+) made from total RNA isolated from Δ*exsD,* Δ*exsD :* p*exsD* under the non-inducing (LB-S) growth condition and NY-4 : p*exsD* under inducing (DMEM) growth condition was used as template for the RT reactions. Mock reactions (−), which did not contain reverse transcriptase, were used as controls against genomic contamination of the RNA preparations.

Because *exsD* in *Pseudomonas* negatively regulates the transcription of T3SS genes, we determined if *V. parahaemolyticus vp1698*, the putative *exsD* homologue, has a similar function. Deletion of *exsD* permitted transcription of T3SS1 genes when bacteria were grown in LB-S, indicating that transcriptional suppression of T3SS1 genes in non-inducing condition is due to the expression of *exsD* (Fig. 4). To determine if T3SS1 genes of the Δ*exsD* strain growing in LB-S are transcribed at levels comparable to the wild-type strain under inducing conditions, we diluted the cDNA templates from NY-4 strain growing in DMEM and Δ*exsD* strain growing in LB-S and tested these dilutions using RT-PCR. The results showed that the abundance of expression of each T3SS1 gene is similar between NY-4 growing in DMEM and Δ*exsD* growing in LB-S, consistent with T3SS1 genes being transcribed at normal levels for the Δ*exsD* strain grown in LB-S (Fig. S1). To control for any polar effects of *exsD* deletion, the Δ*exsD* strain was provided with a wild-type *exsD*-6xHis gene *in trans* and the ectopic expression of ExsD was confirmed by Western blot (Fig. 2B, lane 4). Complementation of Δ*exsD* strain with a wild-type *exsD* restored inhibition of T3SS1 gene transcription in non-inducing conditions, indicating that expression of ExsD is sufficient to repress the transcription of T3SS1 genes in non-inducing condition (Fig. 4). Ectopic expression of ExsD in the wild-type strain prevented transcription of T3SS1 genes when the bacteria were grown in DMEM. Furthermore, ectopic expression of ExsD significantly reduced the ability of *V. parahaemolyticus* wild-type strain to induce host cell death (Fig. 2A and F). These results demonstrated that ExsD is a negative regulator of T3SS1 gene transcription in *V. parahaemolyticus*.

### ExsA but not ExsD binds the promoter sequences of *vp1668* and *vp1687*

**Fig. 5:**
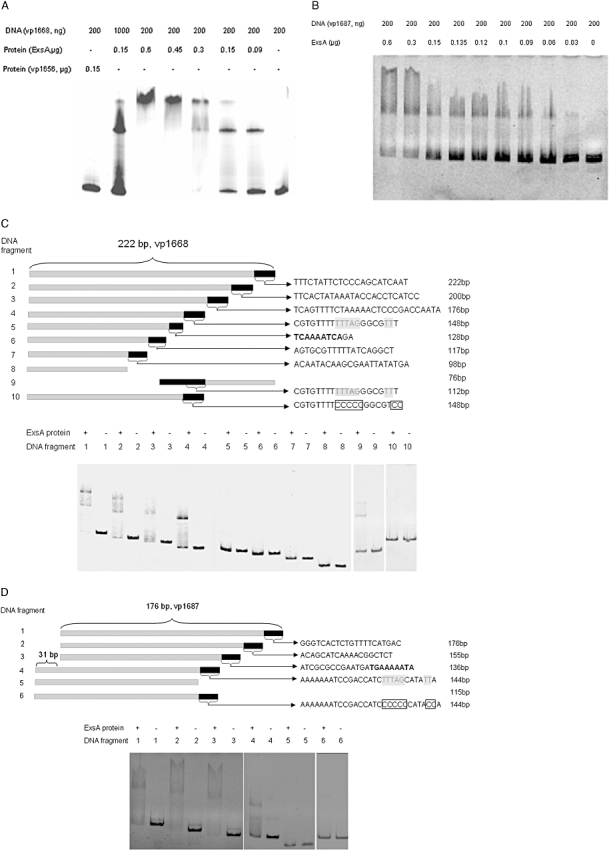
ExsA binds the promoter regions of *vp1668* and *vp1687*. A. Gel shift analysis using ∼180 bp of DNA sequence from the putative promoter region of *vp1668* and purified ExsA. B. Gel shift analysis using ∼180 bp of DNA sequence from the putative promoter region of *vp1687* and purified ExsA. C. Diagram of the DNA fragments within the promoter region of *vp1668* in which the DNA sequences are shown for the black bar; similar sequences to the ExsA binding motif in *Pseudomonas* are shown in bold and putative ExsA binding motif in *V. parahaemolyticus* is underlined; potential ExsA binding sequences are replaced with unrelated boxed sequences (upper panel); Gel shift analysis using each DNA fragment and purified ExsA (0.2 μg) (lower panel). D. Diagram of the DNA fragments within the promoter region of *vp1687* in which the DNA sequences are shown for the black bar; similar sequences to the ExsA binding motif in *Pseudomonas* are shown in bold and a putative ExsA binding motif in *V. parahaemolyticus* is underlined; potential ExsA binding sequences are replaced with unrelated boxed sequences (upper panel); Gel shift analysis using each DNA fragment and purified ExsA (0.3 μg) (lower panel).

We determined if purified proteins ExsA and ExsD were able to bind the promoter sequences of T3SS1 genes. We selected promoter regions upstream of *vp1668* and *vp1687* because these two regions have hypothetical binding motifs. His-tagged recombinant ExsA, ExsD and Vp1656 proteins were purified to apparent homogeneity as shown by SDS-PAGE and Coomassie brilliant blue staining (Fig. S2). Increasing amounts of protein ExsA and ExsD were mixed with ∼180 bp PCR fragment encompassing the putative promoter region of *vp1668* and *vp1687*. Mixtures were size-fractionated by native acrylamide gel eletrophoresis, followed by ethidium bromide staining and visualization. At lower concentrations of ExsA, a low-molecular-weight shifted band was observed while at higher concentrations of ExsA, another high-molecular-weight complex was detected for the promoters of *vp1668* and *vp1687* (Fig. 5A and B). The negative control protein, Vp1656, did not bind the promoter of *vp1668* (Fig. 5A). Furthermore, when excessive DNA probe was used, a shifted band as well as an unshifted band (Fig. 5A) was detected, indicating that binding can be saturated.

To determine if ExsA binds to specific motif(s) within these two regions, DNA fragments with ∼20 bp truncations at the 3′ end (Fig. 5C and D, upper panel) were amplified and subjected to gel mobility shift assay. For promoter *vp1668*, DNA fragments 1–4 have the ability to bind ExsA while fragments 5–8 lose the ability to bind ExsA (Fig. 5C), indicating that at least a portion of the last 22 bp segment of fragment 4 is necessary for ExsA binding. For promoter *vp1687*, DNA fragments 1–4 bind ExsA while fragment 5 does not bind ExsA (Fig. 5D), indicating that at least a portion of the last 29 bp segment of fragment 4 is necessary for binding ExsA. To exclude the possibility that product length affects the ability of ExsA to bind fragment 5 from *vp1668* (128 bp), we generated a shorter DNA fragment 9 (112 bp) from 3′ end of the promoter region encompassing the last 22 bp segment of fragment 4. The results showed that fragment 9 is able to bind ExsA (Fig. 5C). Thus, loss of binding is not related to the product length. Because the last 22 bp of fragment 4 in *vp1668* and the last 29 bp of fragment 4 in *vp1687* share a consensus sequence, TTTAGN_4_TT, we replaced TTTAGGGCGTTT with CCCCCGGCGTCC (fragment 10) in *vp1668* and replaced TTTAGCATATT with CCCCCCATACC (fragment 6) in *vp1687*. The results showed that both fragment 10 of *vp1668* and fragment 6 of *vp1687* lost the ability to bind ExsA (Fig. 5C and D), indicating that TTTAGN_4_TT is the probable ExsA binding motif. In addition, when we used a single concentration of ExsA, two shifted bands appeared in fragments 1 and 2 (possibly 3) of promoter *vp1668* while the shifted band with higher molecular weight disappeared for fragments 3–8 (Fig. 5C). When we tested fragment 9, the higher molecular weight band is again evident. These data strongly suggest the presence of a second ExsA binding motif that is independent of the motif described above. ExsD was mixed with promoter *vp1668* and *vp1687* and no shifted bands were observed (Fig. S3A and S3B), indicating that ExsD does not directly bind the promoter regions of *vp1668* and *vp1687*.

### Activity of promoter *vp1668* and promoter *exsA* increases with inducing conditions

**Fig. 6:**
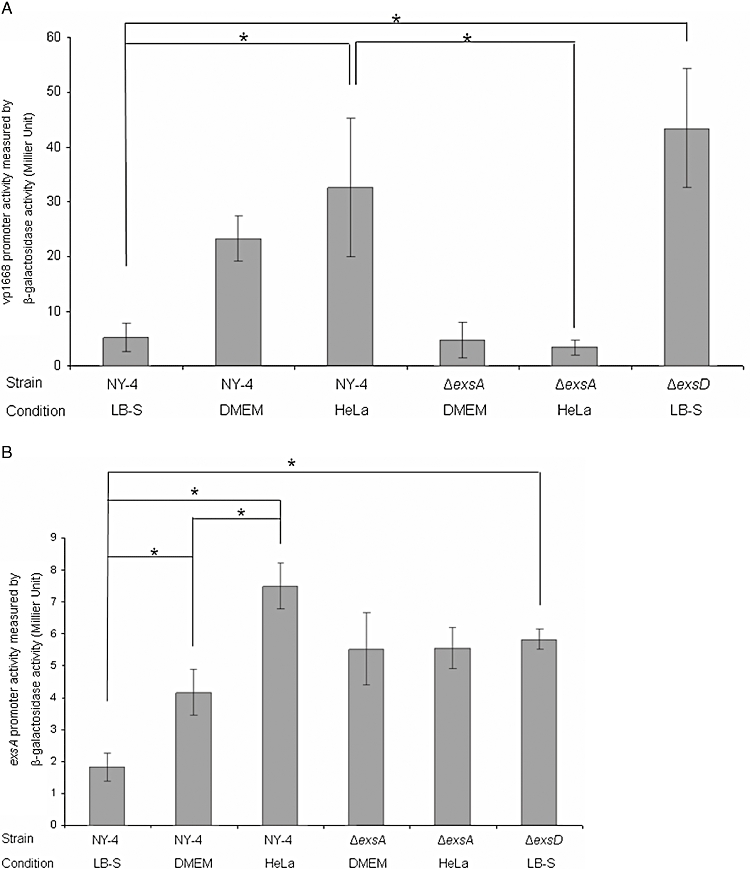
Promoter activity of (A) *vp1668* and (B) *exsA* in different strains and conditions. Bacteria were grown in LB-S, DMEM or contact with HeLa cells for 4 h and levels of β-galactosidase activity were measured (*y*-axis). The values represent the means ± SD of three replicates. Asterisks represent statistical difference (*P* < 0.05).

We characterized the promoter activity of *vp1668* and *exsA* in different growth conditions and different strains. For unknown reasons, we were unable to generate the single copy of transcriptional *lacZ* reporter fusion in the bacterial chromosome for promoter *vp1687*. A single copy of promoters *vp1668–lacZ* and *exsA–lacZ* was introduced into wild-type, Δ*exsA* and Δ*exsD* strains of *V. parahaemolyticus*, and β-galactosidase activities were measured. The activity of the promoter *vp1668* increased five to six times when the wild-type strain of *V. parahaemolyticus* was grown in DMEM or after contact with HeLa cells respectively, compared with bacteria grown in LB-S (Fig. 6A). The activity of the promoter *exsA* increased three to four times when wild-type strain of *V. parahaemolyticus* was grown in DMEM or contact with HeLa cells respectively, compared with bacteria grown in LB-S (Fig. 6B). These results indicate that the promoter activity of *vp1668* and *exsA* increases when the bacteria are grown in DMEM, and that contact with eukaryotic cells produces an additive effect. Promoter activity of *vp1668* in Δ*exsA* strain was about five times less than the wild-type strain when bacteria were grown in DMEM, suggesting that ExsA is crucial for activating promoter *vp1668*. Contact with HeLa cells did not increase the promoter activity of *vp1668* in the Δ*exsA* strain (Fig. 6A), indicating that eukaryotic contact does not override the requirement of ExsA for the activation of promoter *vp1668*. The activity of promoter *vp1668* in Δ*exsD* strain increased approximately eight times compared with the wild-type strain when the bacteria were grown in LB-S (Fig. 6A), verifying that promoter activity of *vp1668* is inhibited by ExsD when bacteria are grown in LB-S (Fig. 4). Promoter activity of *exsA* in the Δ*exsA* strain is similar to that of the wild-type strain when bacteria were grown in DMEM or contact with HeLa cells (Fig. 6B), indicating that *exsA* transcription is not affected by ExsA. Finally, promoter activity of *exsA* in Δ*exsD* strain increased about three times compared with that of the wild-type strain when bacteria were grown in LB-S, confirming an inhibitory role of ExsD in the expression of ExsA (Fig. 6B).

### Expression and secretion of Vp1656 is regulated by ExsA and ExsD

**Fig. 7:**
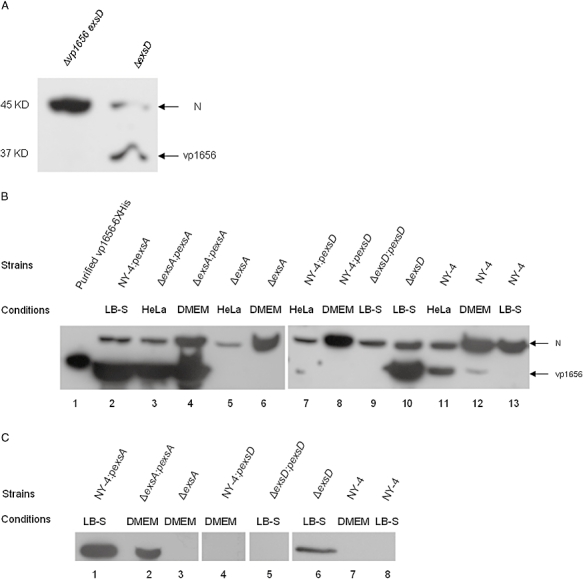
A. Western blot showing the specificity for the polyclonal antibody against Vp1656 for the Δ*exsD* strain (right lane) and the Δ*vp1656 exsD* strain (left lane). A non-specific protein band (‘N’) was detected with this antisera and serves as a positive detection control in these experiments. B. Expression of Vp1656 after bacteria were grown for 2 h in different conditions. Lane 1 shows purified protein Vp1656-6xHis. C. Secretion of Vp1656 after bacteria were grown for 2 h in different conditions.

Vp1656 was shown previously to be secreted by T3SS1 of *V. parahaemolyticus* (Park *et al*., 2004; Ono *et al*. 2006), so we determined if the expression and secretion of Vp1656 were regulated by ExsA and ExsD. Polyclonal antibody against Vp1656 was generated by immunizing mice with the recombinant protein Vp1656 and the specificity of the polyclonal antibody was analysed by Western blot (Fig. 7A). Two bands were detected in the Δ*exsD* strain growing in LB-S while the lower band disappeared in the Δ*vp1656 exsD* mutant strain, consistent with the lower band being Vp1656 as specifically recognized by the polyclonal antibody while the upper band is a non-specific protein (Fig. 7A). After the NY-4 strain was grown in DMEM for 2 h, Vp1656 was expressed (Fig. 7B, lane 12) while Vp1656 was not expressed when bacteria were grown in LB-S (Fig. 7B, lane 13). Furthermore, expression of Vp1656 was increased after contact of wild-type strain with HeLa cells for 2 h compared with grown in DMEM (Fig. 7B, lane 11), confirming that eukaryotic cell contact acts in an additive manner on expression of Vp1656. Interestingly, Vp1656 was not detected in the wild-type strain after 4 h of incubation in both DMEM and LB-S (data not shown). We were not able to detect Vp1656 in the supernatant for the wild-type strain (Fig. 7C, lane 7 and 8). This is possibly due to insufficient assay sensitivity for these conditions. Vp1656 was not detected when the Δ*exsA* strain was grown in DMEM for 2 h (Fig. 7B, lane 6; Fig. 7C, lane 3), or after contact with HeLa cells (Fig. 7B, lane 5). These results indicate that ExsA is required for the expression of Vp1656 in DMEM and eukaryotic cell contact does not override the requirement of ExsA for the expression of Vp1656. Ectopic expression of ExsA in the Δ*exsA* strain and wild-type strain resulted in significant expression of Vp1656 (Fig. 7B, lanes 1–4; Fig. 7C, lanes 1 and 2). Thus, ExsA is sufficient for the expression and secretion of Vp1656 even when bacteria were grown in the non-inducing condition. Vp1656 was expressed and secreted in Δ*exsD* strain (Fig. 7B, lane 10; Fig. 7C, lane 6) and complementation of Δ*exsD* strain with a wild-type *exsD* gene *in trans* prevented Vp1656 expression and secretion when bacteria were grown in LB-S (Fig. 7B, lane 9; Fig. 7C, lane 5). The wild-type strain provided with an *exsD* gene *in trans* blocked Vp1656 expression and secretion when bacteria were grown in DMEM or in contact with HeLa cells (Fig. 7B, lanes 7 and 8; Fig. 7C, lane 4). Thus, ExsD is sufficient to repress the expression and secretion of Vp1656 and eukaryotic cell contact does not override the inhibitory role of ExsD.

### Expression of T3SS1 genes in the absence of ExsD is ExsA-dependent

**Fig. 8:**
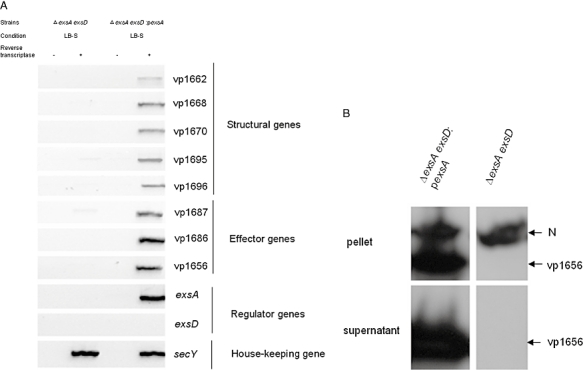
Expression of T3SS1 genes in the absence of ExsD is dependent on ExsA. A. RT-PCR showing that T3SS1 genes are not transcribed in the Δ*exsA exsD* strain while transcription of T3SS1 genes is restored in Δ*exsA exsD* : p*exsA* strain growing in LB-S. Mock reactions (−), which did not contain reverse transcriptase in the RT reaction, were used as controls against genomic contamination of the RNA preparations. *SecY* was a house-keeping gene used as an internal control to ensure that RNA was present in all samples. B. Western blot showing expression and secretion of Vp1656 for the double mutant strain Δ*exsA exsD* and Δ*exsA exsD* strain complemented with a wild-type *exsA* gene *in trans* when grown in LB-S for 4 h. Vp1656 was detected with polyclonal anti-Vp1656 antibody and ‘N’ in the upper panel represents a non-specific band that serves as a positive detection control.

ExsA is required for the expression of T3SS1 genes in the wild-type strain grown under inducing conditions or after contact with HeLa cells (Figs 3 and 7). We next determined if ExsA is also required for the *exsD* mutant strain to express T3SS1 genes when bacteria were grown in non-inducing condition. T3SS1 genes were not transcribed in a Δ*exsA exsD* double mutant strain growing in LB-S (Fig. 8A), while transcription of T3SS1 genes was detected when Δ*exsA exsD* double mutant strain was provided with a wild-type *exsA* gene *in trans*. As expected, transcription of *exsD* gene was not detected for both double mutant strain and the complemented strain (Fig. 8A). After the Δ*exsA exsD* double mutant strain was grown in LB-S, Vp1656 was not expressed (Fig. 8B, upper right panel) or secreted (Fig. 8B, lower right panel), while addition of the double mutant strain with a wild-type *exsA* gene *in trans* restored the expression (Fig. 8B, upper left panel) and secretion of Vp1656 (Fig. 8B, lower left panel). These results indicated that expression of T3SS1 genes by Δ*exsD* strain under non-inducing condition is dependent on ExsA. These results also indicated that transcriptional activity of *exsA* is independent of ExsD.

## Discussion

Transcription and expression of T3SS genes are controlled by specific environmental conditions and a subsequent regulatory cascade. Signals that induce transcription include contact between bacteria and eukaryotic cells or other specific growth conditions. For example, transcription and expression of T3SS genes in *Pseudomonas* and *Yersinia* are activated in response to low concentration of calcium (Finck-Barbancon *et al*., 1997; Vallis *et al*., 1999; Jackson *et al*., 2004) while in *Salmonella*, T3SS genes are activated in high-osmolarity and low-oxygen conditions (Lee and Falkow, 1990; Bajaj *et al*., 1996; Gunn *et al*., 1996; Arricau *et al*., 1998). Co-ordinate regulation of T3SS genes ensures that these genes are properly expressed and fully functional during infection; presumably they are not expressed in other environmental conditions to conserve energy. A previous study showed that secretion of protein can be induced when *V. parahaemolyticus* was cultured in DMEM (Kodama *et al*., 2007); thus we reasoned that DMEM culture conditions activate the transcription of T3SS1 genes. In this study, we determined transcription patterns for 10 T3SS1 genes in *V. parahaemolyticus* of which five genes encode apparatus proteins, three genes encode T3SS substrates, and two genes encode regulatory proteins. We showed that when LB-S was used as growth medium, there is minimal transcription of these genes in the wild-type strain NY-4. *ExsD* is transcribed in LB-S at what appears to be low levels (Fig. 1). When bacteria were grown in tissue culture medium (DMEM), all of the T3SS1 genes tested here were transcribed. For other bacteria, others have shown that tissue culture medium (DMEM) activates a variety of virulence genes including bundle-forming pili (Puente *et al*., 1996), EspA and EspB proteins (Ebel *et al*., 1996) and Tir and Intimin (Shin *et al*., 2001; Abe *et al*., 2002). In one study, investigators demonstrated that NaHCO_3_ in DMEM was responsible for the activation of EspA and EspB proteins in *Escherichia coli* (Abe *et al*., 2002). The specific factor(s) responsible for the activation of T3SS1 gene transcription in *V. parahaemolyticus* remain to be determined.

Mutational analysis demonstrated that environmental regulation of T3SS1 genes in *V. parahaemolyticus* is through the positive regulator, ExsA, and negative regulator, ExsD. ExsA is a member of AraC family of transcriptional activators that are defined by a 100-amino-acid segment of sequence that corresponds to a DNA binding domain of two helix-turn-helix DNA binding motifs (Gallegos *et al*., 1997). Proteins belonging to AraC family regulate many processes in bacteria, including sugar (Mota *et al*., 1999; Plumbridge and Pellegrini, 2004; Raposo *et al*., 2004), amino acid (Zeng and Jin, 2003; Ma *et al*., 2004) and alcohol degradation (Onaca *et al*., 2007), stress responses (van der Straaten *et al*., 2004) and pathogenesis (DiRita, 1992; Gallegos *et al*., 1997; Egan, 2002). Virulence-associated AraC-type proteins regulate gene expression in response to environmental conditions, such as temperature, osmolarity, pH and metal ions (Gallegos *et al*., 1993; 1997). Some of the AraC-type regulators control gene expression by direct interaction with specific molecules. For example, TxtR, an AraC regulator in *Streptomyces,* activates thaxtomin biosynthesis gene expression by binding with cellobiose, an inducer of thaxtomin production (Joshi *et al*., 2007). Another example is UreR protein of *Providencia stuartii* that binds urea directly, which significantly increases the affinity of the protein for its DNA binding sites (Thomas and Collins, 1999; Gendlina *et al*., 2002). Other AraC proteins regulate gene expression by binding directly the promoter region of target genes. In *Pseudomonas*, ExsA activates the transcription of T3SS genes by directly binding to the promoter sequences of T3SS genes in the calcium-limited condition and the consensus binding site within the promoter sequences are TNAAANA, which is centered ∼15 base pairs upstream of the −35 RNA polymerase binding sites (Yahr and Frank, 1994; Hovey and Frank, 1995; Yahr *et al*., 1995).

Sequence analysis of the genomic island of T3SS1 genes in *V. parahaemolyticus* indicated that there were several intergenic spaces that might serve as promoter regions. The first region is between *vp1667* and *vp1668,* and this region may serve as a promoter to initiate the transcription of genes downstream of *vp1667* and *vp1668* because *vp1667* and *vp1668* are transcribed in the opposite directions. The second region is between *vp1687* and *vp1688* and this region may serve as promoter to initiate the transcription of genes downstream of *vp1687*. The third region is between *vp1694* and *vp1695* and the fourth region is between *vp1698* and *vp1699*. Further analysis indicated that putative promoter regions for *vp1668* and *vp1687* contain the DNA sequence of TCAAAATCA and TGAAAAATA respectively, consistent to the ExsA protein binding motif, TNAAANA in *Pseudomonas*. Electrophoretic mobility shift assay (EMSA) results (Fig. 5A and B) demonstrated that purified ExsA protein is able to directly bind these two DNA regions. Surprisingly, DNA fragment 5 from the *vp1668* promoter region, which encompasses a putative binding motif (TCAAAATCA), but does not bind ExsA (Fig. 5C). DNA fragment 4 from the *vp1687* promoter does not encompass its putative binding motif (TGAAAAATA), yet this fragment binds ExsA (Fig. 5D). Further analysis between the last 20 residues of fragment 4 within the promoter region of *vp1668* (Fig. 5C, upper panel) and the last 29 residues of fragment 4 within the promoter region of *vp1687* (Fig. 5D, upper panel) identified a consensus sequence TTTAGN_4_TT. Mutation of this consensus sequence blocks ExsA binding, and thus the consensus sequence encompasses a unique binding motif. Binding of ExsA within the promoter region results in the transcription and expression of Vp1668, Vp1687 and their downstream genes, because transcription of T3SS1 genes and expression of Vp1656 were blocked in the Δ*exsA* strain while addition of *exsA in trans* restored T3SS1 transcription and Vp1656 expression (Figs 3 and 7). Promoter activity of *vp1668* in Δ*exsA* strain decreased to the baseline, indicating that the promoter activity of *vp1668* requires the expression of ExsA.

In *Pseudomonas*, ExsD is a cytosolic protein that directly interacts with ExsA and inhibits ExsA-dependent transcription (McCaw *et al*., 2002). Deletion of ExsD allows transcription of T3SS genes in *Pseudomonas*. We found that under non-inducing condition, only *exsD* was clearly transcribed in *V. parahaemolyticus* (Fig. 1), consistent with ExsD repression of T3SS1 genes. We demonstrated that transcription of T3SS1 genes (Fig. 4) and expression of Vp1656 (Fig. 7B, lane 10; Fig. 7C, lane 6) occurred in the Δ*exsD* strain when bacteria were grown in non-inducing conditions. Furthermore, promoter activity of *vp1668* and *exsA* was significantly increased in Δ*exsD* strain compared with the wild-type strain grown in non-inducing conditions (Fig. 6A and B). These results demonstrated that *V. parahaemolyticus* ExsD functions as an inhibitor of T3SS1 gene expression. With inducing conditions, overexpression of ExsD in wild-type strain inhibited the transcription of T3SS1 genes (Fig. 4). Furthermore, overexpression of ExsD inhibited the expression of Vp1656 when bacteria were grown in DMEM or while in contact with eukaryotic cells (Fig. 7B, lanes 7 and 8). These results demonstrate that *V. parahaemolyticus* ExsD alone is sufficient to block transcription and expression of T3SS1 genes. Because ExsD is not able to bind the promoter of *vp1668* and *vp1687* (Fig. S3A and S3B), it is unlikely that ExsD regulates the transcription of T3SS1 genes directly. Furthermore, when grown in LB-S, the activity of the *exsA* promoter in the wild-type strain is significantly less than *exsA* promoter activity in the Δ*exsD* strain (Fig. 6B), In this situation, there are not environmental signals for transcription of *exsA* and, therefore, this is clear evidence that ExsD blocks transcription of *exsA* [probably in conjunction with another interacting protein(s)].

One interesting finding is that transcription of *exsD* is lower in non-inducing condition than in inducing condition (Fig. 1). In addition, transcription of *exsD* is lower in the Δ*exsA* strain compared with the strain complemented with a wild-type *exsA* gene (Fig. 3). These results indicate that expression of ExsA facilitates the transcription of *exsD* and expression of ExsD blocks transcription of *exsA* (Fig. 4). This appears to be a negative feedback system that modulates transcription and expression of T3SS1 genes. *Pseudomonas* has two additional T3SS gene regulators, *exsC* and *exsE* (Yahr and Wolfgang, 2006). ExsC is able to bind ExsD and prevent binding of ExsD with ExsA and thereby permit T3SS gene transcription (Dasgupta *et al*., 2004). ExsE, a substrate of T3SS, accumulates within the bacterium when bacteria are grown in non-inducing condition and binds ExsC (Urbanowski *et al*., 2005). Binding of ExsE with ExsC allows ExsD to bind ExsA, leading to the transcriptional repression of T3SS genes (Urbanowski *et al*., 2005). Sequence analysis showed that *vp1701* in *V. parahaemolyticus* shares 29% similarity with *exsC* in *Pseudomonas*, and thus it would be interesting to determine if *vp1701* is also involved in the regulation of T3SS1 genes.

We have also found that expression of Vp1656 in wild-type strain was only observed when bacteria were incubated for 2 h while after 4 h of incubation, expression of Vp1656 was not detectable. This is consistent with an earlier report showing that quorum sensing negatively regulates the expression of Vp1656 (Henke and Bassler, 2004). Vp1656 is produced and secreted only when quorum sensing was knocked out (Henke and Bassler, 2004). Our study showed that deletion of *exsD* resulted in the constitutive expression and secretion of Vp1656, suggesting a possible interaction between *exsD* with quorum sensing. Furthermore, overexpression of ExsA also led to the constitutive expression and secretion of Vp1656, indicating that ExsA might play a role in the repression of quorum sensing in *V. parahaemolyticus*. Regulation of quorum sensing by AraC proteins has been observed in *Pseudomonas aeruginosa* (Dong *et al*. 2005) and *V. parahaemolyticus* (Enos-Berlage *et al*. 2005), and here we suggest another possible mechanism to regulate quorum sensing via the *exsA* gene in *V. parahaemolyticus*. We also found that contact with eukaryotic cells increased the expression of Vp1656 in an additive manner compared with growth in DMEM alone.

Based on our results, it appears that when *V. parahaemolyticus* is grown in non-inducing condition (e.g. LB-S), ExsD is expressed and blocks transcription of *exsA*. When *V. parahaemolyticus* is grown under inducing conditions (e.g. DMEM), *exsA* is transcribed by an environmental signalling pathway and ExsA binds directly to the promoter region of T3SS1 genes, leading to their expression. At the same time, ExsA increases the transcription of *exsD* (Fig. 3), which in turn provides a negative feedback to downregulate and thereby modulate the expression of T3SS1 genes. Our results suggest that the most parsimonious pathway by which ExsD exerts its effects is by blocking transcription of *exsA* rather than by blocking the environmental signalling pathway or by blocking transcription of individual T3SS1 genes.

In summary, we identified conditions that induce the expression of T3SS1 genes of *V. parahaemolyticus* and showed that ExsA activates their expression by directly binding the promoter region of T3SS1 genes. In addition, transcription of T3SS1 genes is blocked by overexpression of ExsD while deletion of *exsD* gene permitted the transcription of T3SS1 genes under non-inducing conditions. Either deletion of *exsA* or overexpression of ExsD attenuates the ability of *V. parahaemolyticus* to induce host cell death.

## Experimental procedures

### Bacterial strains, plasmids and growth conditions

The strains used in this study are listed in Table 1. All *V. parahaemolyticus* strains were derived from the wild-type strain NY-4 (X. Zhou, M.E. Konkel, and D.R. Call, submitted). Strains were grown in LB broth or LB agar supplemented with 2.5% sodium chloride at 37°C. *E. coli* S17 λ*pir* was used in gene deletion experiments and was cultured in LB medium. Plasmid pMMB207 was used in complementation experiments and plasmid pDM4 was used for gene knockout experiments. Other derivative plasmids are listed in Table 1. When appropriate, antibiotics were added at the following concentrations: ampicillin, 100 μg ml^−1^; chloramphenicol, 25 μg ml^−1^ for *E. coli* and 5 μg ml^−1^ for *V. parahaemolyticus*.

**Table 1:** Strains and plasmids used in this study.

Strains and plasmids	Descriptions	Sources
*E. coli*		
S17-1λ*pir*	*thi pro hsdR hsdM*^+^*recA* RP4-2-Tc::Mu-Km::Tn*7*λ*pir*	Milton *et al*. (1992)
S17-*exsA–lacZ*	S17 carrying pNK8-*exsA–lacZ*	This study
S17-1668*–lacZ*	S17 carrying pNK8-1668–lacZ	This study
S17-pMMB207-RBS-*exsA*	S17 carrying pMMB207-RBS-*exsA*	This study
S17-pMMB207-*exsD*	S17 carrying pMMB207-*exsD*	This study
S17-pDM4-1699-1F + 2R	S17 carrying pDM4-1699-1F + 2R	This study
S17-pDM4-1698-1F + 2R	S17 carrying pDM4-1698-1F + 2R	This study
*V parahaemolyticus*		
NY-4	Clinical isolate O3 : K6	
Δ*exsA*	*exsA* (*vp1699*) deletion mutant	This study
Δ*exsA* : *pexsA*	Δ*exsA* complemented with *exsA* gene located in the plasmid of pMMB207	This study
NY-4 : p*exsA*	Wild-type strain containing *exsA* gene located in the plasmid of pMMB207	This study
*vp1668–lacZ*::NY-4	Promoter sequences of *vp1668* was inserted in the upstream of *lacZ* gene in NY-4 strain	This study
*vp1668–lacZ*::Δ*exsA*	Promoter sequences of vp1668 was inserted in the upstream of lacZ gene in Δ*exsA* strain	This study
*vp1668–lacZ*::Δ*exsD*	Promoter sequences of *vp1668* was inserted in the upstream of *lacZ* gene in Δ*exsD* strain	This study
*vp1699–lacZ*::NY-4	Promoter sequences of *vp1699* was inserted in the upstream of *lacZ* gene in NY-4 strain	This study
*vp1699–lacZ*::Δ*exsA*	Promoter sequences of *vp1699* was inserted in the upstream of *lacZ* gene in Δ*exsA* strain	This study
*vp1699–lacZ*::Δ*exsD*	Promoter sequences of *vp1699* was inserted in the upstream of *lacZ* gene in Δ*exsD* strain	This study
Δ*exsD*	*exsD (vp1698*) deletion mutant	This study
Δ*exsD* : p*exsD*	Δ*exsD* complemented with *exsD* gene located in the plasmid of pMMB207	This study
NY-4 : p*exsD*	Wild-type strain containing *exsD* gene located in the plasmid of pMMB207	This study
Δ*exsA exsD*	*exsA* and *exsD* double mutant strain	This study
Δ*exsA exsD* : p*exsA*	Δ*exsA exsD* complemented with *exsA* gene located in the plasmid of pMMB207	This study
Δ*vp1656 exsD*	Insertional mutation of *vp1656* on the background of Δ*exsD* strain	This study
*Yersenia enterocolitica*		
JB580v	Serogroup O : 8, Nal^r^Δ*yenR* (r^-^ m^+^)	Young and Young (2002)
Plasmids		
pMMB207	RSF1010 derivative, *IncQ lacI*^q^ Cm^r^ P*tac oriT*	Morales *et al*. (1991)
pNK8	MobRP4 oriR6K, transcriptional reporter vector	Walker and Miller (2004)
pDM4	A suicide vector with ori R6K *sacB*; Cm^r^	Milton *et al*. (1996)
pMMB207-RBS-*exsA*	*exsA* coding sequences and the sequences for 6 His amino acids at the C-terminus cloned into pMMB207	This study
pMMB207-*exsD*	*exsD* coding sequences and the sequences for 6 His amino acids at the C-terminus cloned into pMMB207	This study
pMMB207-1656	Vp1656 coding sequences and the sequences for 6 His amino acids at the C-terminus cloned into pMMB207	This study
pNK8-*exsA–lacZ*	Promoter sequences of *exsA* cloned into pNK8	This study
pNK8-1668–lacZ	Promoter sequences of *vp1668* cloned into pNK8	This study
pDM4-1699-1F + 2R	Flanking region sequences of *exsA* cloned into pDM4	This study
pDM4-1699-1F + 2R	Flanking region sequences of *exsD* cloned into pDM4	This study
pDM4-1656insertion	Internal region of *vp1656* cloned into pDM4	This study

### Construction of deletion mutants

Deletion mutants were generated by homologous recombination. A chromosomal deletion in the *exsA* gene wasconstructed by allelic exchange using a suicide vector, pDM4, carrying DNA fragments corresponding to *exsA* flanking regions (Milton *et al*., 1996). Two DNA fragments were amplified by PCR with *V. parahaemolyticus* strain NY-4 chromosomal DNA as a template with primer pairs 1699-1F and 1699-1R and 1699-2F and 1699-2R respectively (Table 2). Fragment 1 (approximately 1000 bp upstream of the start codon) amplified with 1699-1F and 1699-1R was digested with XhoI and BglII and fragment 2 (approximately 1000 bp downstream of the stop codon) amplified with 1699-2F and 1699-2R was digested with BglII and XbaI. These two digested fragments were ligated into the pDM4 suicide vector, which had been digested with XhoI and XbaI. The resultant plasmid was designated pDM4-1699-1F + 2R. This plasmid was transformed into *E. coli* S17 λ*pir*, resulting in the strain S17-pDM4-1699-1F + 2R. pDM4-1699-1F + 2R was then transferred into the *V. parahaemolyticus* NY-4 wild-type strain by conjugation, and both ampicillin- and chloramphenicol-resistant transconjugants were selected. Ampicillin was used to select against *E. coli* and chloramphenicol was used to select for transconjugants. To complete the allelic exchange, the integrated suicide plasmid was induced to excise from the chromosome by growth on LB-S medium containing 5% sucrose. Chloramphenicol-sensitive clones were obtained and screened by PCR with primers 1699-up and 1699-down that lie adjacent to the *vp1699*. One clone was selected with the *exsA* gene deletion and designated as Δ*exsA*. Construction of *exsD* deletion mutant was performed in the same manner by using the following primers: 16981-F, 1698-1R, 1698-2F, 1698-2R, 1698-up and 1698-down (Table 2). One PCR-confirmed clone was isolated and designated as Δ*exsD*. Double mutant strain was generated by conjugating the plasmid pDM4-1699-1F + 2R from *E. coli* S17 λ*pir* to Δ*exsD* strain and one PCR-confirmed clone was isolated and designated as Δ*exsA exsD*. For construction of Δ*vp1656 exsD*, primers1656insertion_FW and 1656insertion_RE were used to amplify the internal region of *vp1656*. PCR fragment was digested with XhoI and BglII and were ligated into pDM4 digested with the same enzyme. The resultant plasmid was designated pDM4-1656insertion and conjugated into Δ*exsD* strain. One PCR-confirmed clone was isolated and designated as Δ*vp1656 exsD.*

**Table 2:** Primers used in this study.

Primer name	Sequences (5′-3′)
1698-1F	AGGATAAACTCGAGCAAACAGCTCATCCGTCGAG
1698-1R	AGTTAGTCTAGAGGGCAGTATATGTTAGATAAAATG
1698-2F	AGTTAGTCTAGACCCTTTCGAACACTCCAGAAT
1698-2R	AGTTAGAGATCTGTAGAAAATGGATGTGTCAGG
1698-up	TTTTCTCCGGCAATAAAAGGCC
1698-down	TTTTGAAAGTCACCGACGGAC
1698-His-up	AGGATAGAATTCTAAGGAGGTAGGATAATAATGCGGAGAAGAACACAAATG
1698-His-down	AGTTAGTCTAGATTAATGGTGATGGTGATGGTGAATCTGGCTGAGATGGTTACAAG
1699-1F	AGGATAAACTCGAGTCGTTATAAACCACGTCACATGC
1699-1R	AGTTAGTCTAGAAATGACTTATGACTAAAATTTTC
1699-2F	AGTTAGTCTAGATTTCTACCCTTCATAATTTTTAATTTC
1699-2R	AGTTAGAGATCTTGTTATTGACTGGATGCGCTC
1699-up	TTCATTTGTGTTCTTCTCCGC
1699-down	CTTTGCTTCTTTAATTGAAATTG
1699-His-up	AGGATAGGATCCTAAGGAGGTAGGATAATAATGGATGTGTCAGGCCAACT
1699-His-down	AGTTAGTCTAGATTAATGGTGATGGTGATGGTGATTCGCGATGGCGACTTG
lacZ-exsA-FW	AGGATATCTAGAGTGATTTATGATTATATCCTACGC
lacZ-exsA-RE	AGTTAGAGATCTTTTCTACCCTTCATAATTTTTAAT
lacZ-1668-FW	AGGATATCTAGAAATACTCAT TCACTTGCACTC
lacZ-1668-RE	AGTTAGAGATCTAATGTAAAAAATATGCGCAATG
Promoter1668-FW	AATACTCATTCACTTGCACTC
Promoter1668-RE	AATGTAAAAAATATGCGCAATG
Promoter1687-FW	CAGAGTAGGGCATCACCG
Promoter1687-RE	TTGAATGAATAATCCTATAGTG
1668FW_motif	CGGACCGATGCATCAAAC
1668RE_motif1	ATTGATGCTGGGAGAATAG
1668RE_motif2	GGATGAGGTGGTATTTATAG
1668RE_motif3	TATTGGTCGGGAGTTTTTAG
1668RE_motif4	AAACGCCCTAAAAAAACACG
1668RE_motif5	GTCTGATTTTGACAGCCTG
1668RE_motif6	CAGCCTGATAAAAACGCAC
1668RE_motif7	TCATATAATTCGCTTGTATTG
1668RE_motif8	ACGAAAGGAGTGCAAGTGA
1668FW_motif9	CAGGCTGTCAAAATCAGACG
1668RE_motif10	GGACGCCGGGGGAAAACACGTCTGATTTTGAC
EMSA1687motif_FW	AAACAACTCAGTGAGCGG
EMSA1687motif1_RE	GTCATGAAAACAGAGTGACC
EMSA1687motif2_RE	AGAGCCGTTTTGATGCTG
EMSA1687motif3_RE	TATTTTTCATCATTCGGCGC
EMSA1687motif4_RE	TAATATGCTAAAGATGGTCGG
EMSA1687motif5_RE	CGTTTTAAGACGTTTGAAAAC
EMSA1687motif6_RE	TGGTATGGGGGGGATGGTCGGATTTTTTTCG
1656insertion_FW	AGGATAAACTCGAGATTAGATGGGCCGAAAGCTC
1656insertion_RE	AGTTAGAGATCTCGAATGTGCACGTTTTACTTG
vp1656-up	AGGATAGAATTCAGGAGATATACCATGTTGGATAAAATTGGTGGAAC
vp1656-down	AGTTAGTCTAGATTAATGGTGATGGTGATGGTGCACTGTCGGGATAGATGCGC
1662–600-up	GTCACGCAAAAAGGCATGAGC
1662–600-down	AAAACGCAGGTGAATTCCCGG
1668–600-up	CAAGAGCCAAGTGCTTGGTATC
1668–600-down	CATGTCGTCACCTTCGACCA
1670–600-up	AGAGCGCTCTCTTGCGTCAC,
1670–600-down	TTTGGGATGTTCATCATTCGGG
1686–600-up	ATTCTAAATGAAGGCAAACTCAGC
1686–600-down	GTTTAAATCCGTACTTGCGAGC
1687–600-up	ATGGCTAATGGATTTATTACCG
1687–600-down	TTACACCCTTAATGTAGAGAATTG
1695–600-up	GGATGTCAGGACGTAACCATC
1695–600-down	CCGTCAGCAGCAACGTGC
1696–600-up	ATGTGGTACTTCGATGGCG
1696–600-down	TTGTGGCTGATCGAGTTGAG
1698-600-up	GTTGTCCACGTGAATCACCGA
1698-600-down	GTAGTCCGCCAGTTCTTTCAA
1699-600-up	GTCGTTCACAATGGTCAATTACG
1699-600-down	CGTCAGCAAAAGCTTGTGTG
secY1	AGGATAAACTCGAGTGGTGCTCTGGAGCGTGCATC
secY2	AGTTAGTCTAGACCTTGTTGACGCTTCGCGTAG

### Complementation

Primers 1699-His-up and 1699-His-down were used to amplify the complete gene of *exsA* with a 6xHis tag added at the C-terminus of the gene. Amplified PCR product was digested with BamHI and XbaI and ligated into the plasmid pMMB207 (digested with the same enzymes), resulting in the plasmid pMMB207-RBS-*exsA*. Plasmid pMMB207-RBS-*exsA* was transformed into *E. coli* S17 λ*pir,* resulting in the strain S17-pMMB207-RBS-*exsA,* and then conjugated from *E. coli* S17 λ*pir* into Δ*exsA*, Δ*exsA exsD* and the *V. parahaemolyticus* NY-4 wild-type strain, resulting in Δ*exsA* : p*exsA*, Δ*exsA exsD* : p*exsA* and NY-4 : p*exsA* respectively. Primers 1698-His-up and 1698-His-down were used to amplify the complete gene of *exsD* with a 6xHis tag added at the C-terminus of the gene. Amplified PCR product was digested with EcoRI and XbaI and ligated into the plasmid pMMB207 digested with the same enzymes, resulting in the plasmid pMMB207-*exsD*. Plasmid pMMB207-*exsD* was transformed into *E. coli* S17 λ*pir* by electroporation and then conjugated from *E. coli* S17 λ*pir* into Δ*exsD* and wild-type NY-4 strain, resulting in Δ*exsD* : p*exsD* and NY-4 : p*exsD* respectively (Table 1).

### Construction of single-copy *lacZ* reporter fusions in the bacterial chromosom

Transcriptional *lacZ* reporter fusions were constructed by cloning the upstream region of *exsA* and *vp1668* into a suicide vector, pKN8. For the *exsA* promoter, approximately 500 bp DNA fragment upstream of *exsA* gene was amplified by PCR using primers lacZ-exsA-FW and lacZ-exsA-RE (Table 2). PCR product was digested with XbaI and BglII and cloned into pNK8 (digested with the same enzymes) resulting in the plasmid pNK8-*exsA–lacZ*. For *vp1668* promoter, approximately 160 bp DNA fragment upstream of *vp1668* was amplified by PCR using primers lacZ-1668-FW and lacZ-1668-RE. PCR product was digested with XbaI and BglII and cloned into pNK8 (digested with the same enzymes), resulting in the plasmid pNK8-1668–lacZ. These two plasmids were transformed into *E. coli* S17 λ*pir* by electroporation, resulting in the strains S17-*exsA–lacZ* and S17-1668–lacZ respectively. pNK8-1668–lacZ and pNK8-*exsA–lacZ* were integrated into *V. parahaemolyticus* strains of NY-4, Δ*exsA* and Δ*exsD* by conjugation, resulting in the *lacZ* transcriptional fusion strains: *vp1668–lacZ*::NY-4, *vp1668–lacZ*::Δ*exsA*, *vp1668–lacZ*::Δ*exsD*, *vp1699–lacZ*::NY-4, *vp1699–lacZ*::Δ*exsA*, *vp1699–lacZ*::Δ*exsD* (Table 1).

### Infection and LDH assay

*Vibrio parahaemolyticus* was harvested from an overnight broth culture and pelleted by centrifugation at ∼6000 *g* r.p.m. at room temperature. The bacterial pellets were re-suspended in DMEM (Invitrogen, Carlsbad, CA) containing 1% (v/v) Fetal Bovine Serum (HyClone, Logan, Utah). Bacterial suspensions (10 μl) were added to the wells of a 12-well plate, where each well contained HeLa cells (10^6^) (CCL-2), to achieve a multiplicity of infection (m.o.i.) of ∼100 cfu per cell. Plates were centrifuged at ∼600 *g* to synchronize bacteria–HeLa host cell contact. For LDH release assay, the FBS concentration in the medium was reduced from 10% to 1% to reduce background LDH activity. Supernatants were collected at 4 h post infection and LDH activity was measured with Cytotoxicity Detection Kit (Promega, Madison, WI) according to manufacturer's instructions. Maximum LDH release was achieved by lysis of cells with lysis buffer provided in the kit. Spontaneous LDH release in the supernatant of uninfected cells was also measured as described. Percentage cytotoxicity was calculated with the formula:

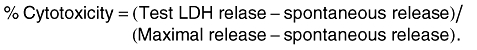


### RNA isolation and RT-PCR

For bacterial growth in DMEM, an overnight culture (1.5 ml) was centrifuged and the pellet was re-suspended in 15 ml of DMEM supplemented with 1% FBS. For bacterial growth in LB-S, overnight culture (0.15 ml) was directly inoculated into 15 ml of LB-S medium. After 3 h incubation, total RNA was isolated by RNA Easy kit (Qiagen, Valencia, CA). Isolated RNA was treated with DNase I for 30 min to remove DNA and was reverse-transcribed into cDNA using 2 μg of RNA, 200 ng of random hexamers and Superscript III (Invitrogen, Carlsbad, CA). PCR was performed according to the manufacturer's instructions (1 U of Master *Taq* polymerase, 200 μM each of the four dNTPs and 1 μM each primer). Primer pairs (the last 22 primers listed in Table 2) amplifying internal fragments were used for semi-quantitative analysis of gene expression. Cycling parameters were identical for all primer sets (Table 2): one cycle of 94°C for 4 min; 30 cycles of 95°C for 1 min, 52°C for 1 min and 72°C for 1 min; and a final incubation at 72°C for 5 min.

### Cloning and expression of *exsA*, *exsD* and *vp1656*

For cloning of *vp1656*, primers vp1656-up and vp1656-down were used to amplify the entire coding sequences along with sequences for 6 His amino acids at the C-terminus. PCR product digested with EcoRI and XbaI was cloned into pMMB207 (digested with the same enzymes), resulting in the plasmid pMMB207-1656. Plasmid pMMB207-1656 was transformed into *E. coli* S17 λ*pir,* resulting in the strain S17-pMMB207-1656, which was conjugated into *Yersinia enterocolitica* for expression. For expression of *exsA* and *exsD*, the plasmids pMMB207-RBS-*exsA* and pMMB207-*exsD* were conjugated from *E. coli* S17 λ*pir* into *Y. enterocolitica*. We used *Y. enterocolitica* to express these proteins because ExsA and ExsD were insoluble when expressed in the heterologous host strain *E. coli* BL21. An overnight culture (20 ml) of *Y. enterocolitica* containing each plasmid was diluted into 500 ml of LB medium and incubated for 2 h with shaking at 26°C before adding 1 mM IPTG. Protein expression was induced for 12 h in 26°C with vigorous shaking before pelletting cells. Pellets were re-suspended in 15 ml of binding buffer (50 mM NaH_2_PO_4_, pH 8.0, 0.5 M NaCl, 10 mM imidazole) and sonicated for 15 min. Insoluble cell fractions were removed by centrifugation at ∼3000 *g* for 10 min and the supernatant were loaded into an Ni^2+^ resin column (Invitrogen). After binding for 1 h, the column was washed 15× with washing buffer (50 mM NaH_2_PO_4_, pH 8.0, 0.5 M NaCl, 50 mM immidazole) before being eluted with elution buffer (50 mM NaH_2_PO_4_, pH 8.0, 0.5 M NaCl, 300 mM immidazole). The concentration of recombinant proteins was determined by measuring OD_280_ with a NanoDrop (NanoDrop Technologies, Wilmington, DE). Purity of the protein samples was analysed by SDS-PAGE and gel staining. Purified protein Vp1656 was submitted to Monoclonal Antibody Center at Washington State University for immunization of two mice to raise polyclonal antibody against Vp1656.

### The EMSA

DNA fragments of approximately 180 bp, corresponding to sequences upstream of *vp1668* and *vp1687* start codons, were amplified by PCR using primers promoter1668-FW/promoter1668-RE and promoter1687-FW/promoter1687-RE. Forward primer for fragements 1–8 in Fig. 5C is 1668FW_motif and the reverse primers are 1668RE_motif1–8 respectively. Fragment 9 in Fig. 5C was amplified with primers 1668FW_motif9 and 1668RE_motif1. Fragment 10 in Fig. 5C was amplified with primers 1668FW_motif and 1668RE_motif10. Forward primer for fragments 1–3 in Fig. 5D is promoter1687-FW and the reverse primers are EMSA1687motif1_RE, EMSA1687motif2_RE and EMSA1687motif3_RE respectively. Forward primer for fragments 4–6 in Fig. 5D is EMSA1687motif_FW and the reverse primers are EMSA1687motif4_RE, EMSA1687motif5_RE and EMSA1687motif6_RE respectively. Increasing amounts of the purified proteins were mixed with 200 ng of DNA probes (PCR products) in buffer containing 10 mM Tris (pH 7.5), 1 mM EDTA, 100 mM KCl, 0.1 mM DTT, 5% v/v glycerol and 0.01 mg ml^−1^ BSA, and binding reactions were incubated at room temperature for 30 min before loading onto a 6% native polyacrylamide gel in 0.5× TBE buffer (45 mM Tris-HCl, 45 mM boric acid, 1 mM EDTA, pH 8.0). DNA–protein complexes were electrophoresed for 70 min at 120 V followed by staining with ethidium bromide and imaging.

### Preparation of proteins and Western blot analysis

To collect extracellular proteins, overnight culture grown in LB-S was diluted into LB-S (1:100) and DMEM (1:10) with addition of antibiotics as necessary. Diluted samples were grown for 2 and 4 h at 37°C with shaking. Protein expression was induced by the addition of IPTG to the final concentration of 1 mM. The cells were removed from 15 ml culture by centrifugation at ∼3000 *g* for 15 min and the supernatant for each sample was passed through a 0.22 μm syringe filter to exclude the remaining bacteria within the supernatant. Trichloroacetic acid was added to the supernatant to achieve a final concentration of 10% (v/v) and incubated on ice overnight to precipitate proteins. The samples were centrifuged at 4°C for 20 min at ∼12 000 *g* followed by washing once with ice-cold acetone and the protein pellets were re-suspended in 50 μl of 1× loading buffer (40 mM Tris/HCl pH 8.0; 5% mercaptoethanol; 1% SDS; 5% glycerin; 1% bromphenol blue). To collect the total proteins for detection of Vp1656, an overnight culture was diluted into LB-S or DMEM and grown for 2 or 4 h with addition of antibiotics and IPTG as needed. The bacterial cultures (15 ml) was centrifuged for 20 min at ∼3000 *g* and the pellets were re-suspended in 500 μl PBS, followed by sonication and the addition of equal volume of 2× loading buffer. To collect proteins for detection of recombinant 6xHis-tagged ExsA and ExsD, an overnight culture for each recombinant strain was diluted 1:100 into LB-S and grown for 6 h before collecting proteins as described above. To collect the proteins from infected HeLa samples, overnight culture was centrifuged and re-suspended into equal volume of DMEM and bacterial suspension (40 μl) was added to each well of 6-well plate containing ∼10^6^ HeLa cells to achieve an m.o.i. of 25 cfu per cell. Plates were centrifuged at 600 *g* to synchronize bacteria–host cell contact. Two or four hours post infection, the infected cells were scraped from the well and re-suspended in 200 μl of PBS. After sonication, an equal volume of 2× loading buffer was added into each sample for SDS-PAGE and Western blot analysis. For Western blot analysis, all the samples were heated at 100°C for 5 min and loaded on a 12% (w/v) polyacrylamide gel. After electrophoresis, proteins were electrophoretically transferred to a nitrocellulose membrane for 12 h. The membrane was blocked with 5% skim milk in PBS containing 0.1% Tween 20 and probed with monoclonal anti-His antibody (1:2000) (Invitrogen) or polyclonal anti-Vp1656 (1:1000) for 1 h at room temperature. The secondary antibody was an anti-mouse immunoglobulin conjugated to horseradish peroxidase and was diluted in PBS with 5% milk at 1:5000. The blots were developed by using the Western blotting kit according to the manufacturer's instructions (Bio-Rad, Hercules, CA).

### β-Galactosidase assay

Overnight culture for each strain was diluted 1:10 into DMEM and 1:100 into LB-S with addition of antibiotics as needed. After 4 h of incubation with shaking, 0.5 ml of the culture was centrifuged and the pellets were re-suspended in PBS (0.5 ml) and permeabilized by adding 50 μl of SDS (0.5%) before measuring OD_600_. To determine the β-galactosidase activity after contact with HeLa cells, overnight culture (0.5 ml) suspended in DMEM medium was added into a 25 m^2^ flask containing confluent adhered HeLa cells (∼10^7^). The flask was centrifuged to synchronize bacteria–HeLa host cell contact. After 2 h incubation, nonadherent as well adherent bacteria were collected by carefully washing the HeLa cells and re-suspending in PBS (0.5 ml). The suspension of the bacteria was permeabilized by adding 50 μl of SDS (0.5%) before measuring OD_600_. O-nitrophenyl-beta-galactoside (4 mg ml^−1^) (200 μl; Sigma, St. Louis, MO) was added to the permeabilized samples and the reaction was incubated until sufficient yellow colour developed before adding stop solution (0.1 ml of 1 M Na_2_CO_3_). OD_414_ for each sample was recorded and β-galactosidase activity was calculated by using the following formula:

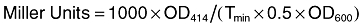


### Statistical analysis

Statistical analysis was performed with anova and *P*-values < 0.05 were considered statistically significant. Pairwise comparisons were conducted using a Tukey–Kramer test and the analyses were conducted using ncss 2004 (Number Cruncher Statistical Systems, Kaysville, UT).

## Supplementary material

This material is available as part of the online article from: http://www.blackwell-synergy.com/doi/abs/10.1111/

j.1365-2958.2008.06326.x

(This link will take you to the article abstract).
