# Supplementary material for: Type III secretion system 1 genes in Vibrio parahaemolyticus are positively regulated by ExsA and negatively regulated by ExsD
Source: Mol Microbiol. 2008 Jun 16;69(3):747–64. doi: 10.1111/j.1365-2958.2008.06326.x (PMC2610376; doi:10.1111/j.1365-2958.2008.06326.x)
Supplement: Supplementary file 1 [file mmi0069-0747-SD1.pdf]

Supplementary Fig. S1. RT-PCR showing that expression of T3SS1 genes are fully restored in Δ*exsD* strain growing in LB-S compared to wild-type strain growing in DMEM. cDNAs (+) made from total RNA isolated from *V. parahaemolyticus* strain NY-4 under DMEM growth condition and Δ*exsD* strain under LB-S growth condition were used as templates for PCR. Mock reactions (-), which did not contain reverse transcriptase in the RT reaction, were used as controls against genomic contamination of the RNA preparations. *SecY* was a house-keeping gene used as an internal control to ensure RNA was present in all samples. cDNA templates were serially diluted (5-fold) and RT-PCR was performed with various dilutions of the templates for each T3SS1 gene.

Supplementary Fig. S2. SDS-PAGE analysis of purified ExsA (lane 1), ExsD (lane 3) and Vp1656 (lane 2). His-tagged proteins ExsA, ExsD and Vp1656 were expressed and purified by using an Ni^{2+} resin column.

Supplementary Fig. S3. ExsD does not bind the promoter region of *vp1668* and *vp1687*. (A) Gel shift analysis using ~180 bp of putative promoter region of *vp1668* probe and purified ExsD; (B) Gel shift analysis using ~180 bp of putative promoter region of *vp1687* probe and purified ExsD. Protein Vp1656 was included as a negative control. The concentration of DNA and protein were shown in the figure.

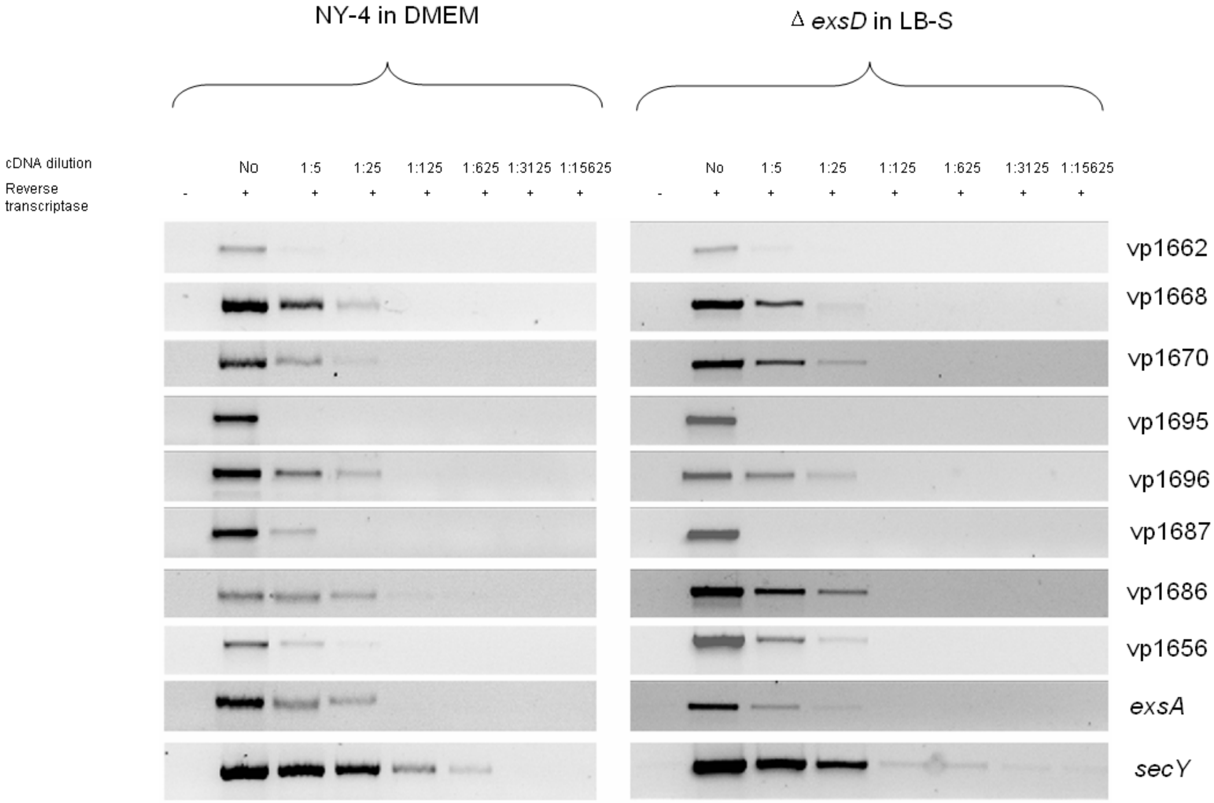

Supplementary Fig. S1

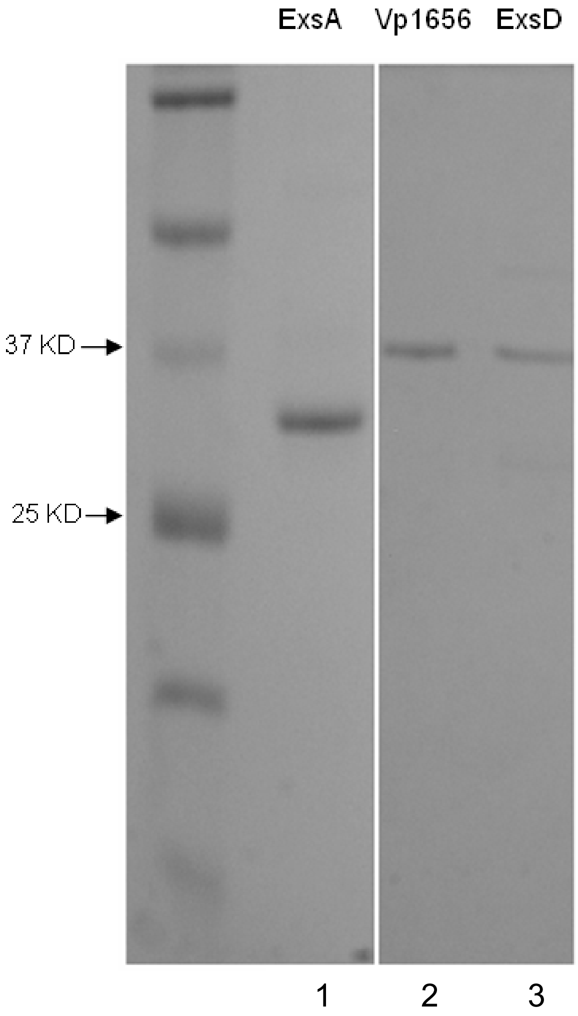

Supplementary Fig. S2

A

DNA (vp1668, ng)	200	1000	200	200	200	200	200	200
Protein (ExsD,µg)	-	0.15	0.6	0.45	0.3	0.15	0.09	-
Protein (vp1656, µg)	0.15	-	-	-	-	-	-	-

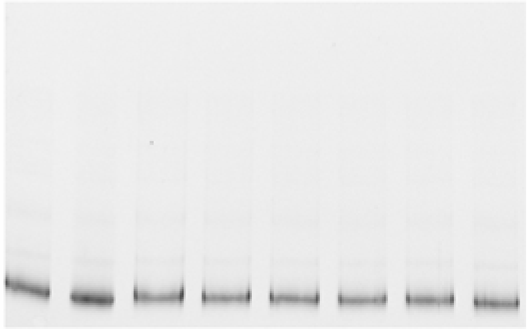

B

DNA (vp1687, ng)	200	1000	200	200	200	200	200	200	200
Protein (ExsD,µg)	-	0.15	0.9	0.6	0.45	0.2	0.15	0.09	-
Protein (vp1656, µg)	0.5	-	-	-	-	-	-	-	-

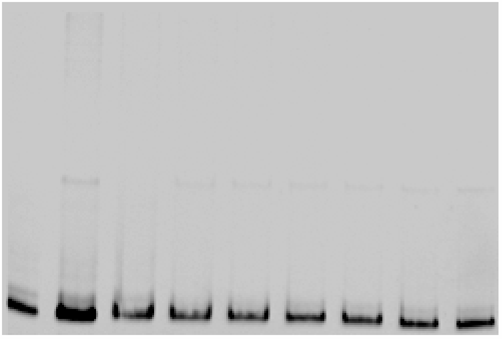

Supplementary Fig. S3
